# 532 nm Q-switched Laser Therapy for Iron Extravasation-Induced Skin Pigmentation: A Case Report and Literature Review

**DOI:** 10.7759/cureus.92113

**Published:** 2025-09-12

**Authors:** Massimo Vitale, Alessandra Zevini, Daniela Martinelli, Riccardo Barini

**Affiliations:** 1 Aesthetic Medicine, Studio Medico Vitale, Bologna, ITA; 2 Medical Affairs, El.En. Group, Calenzano, ITA

**Keywords:** case report, cutaneous siderosis, iron extravasation, q-switched nd:yag laser treatment, skin pigmentation

## Abstract

Cutaneous siderosis represents a challenging dermatological condition characterized by iron deposition within the skin, resulting in persistent brownish hyperpigmentation that can significantly impact a patient's quality of life. While this condition can arise from various etiologies, intravenous iron infusions remain a common cause due to extravasation and subsequent iron deposition in cutaneous tissues. This case report explores the efficacy of Q-switched 532 nm Nd:YAG laser treatment for cutaneous siderosis, a wavelength selected after demonstrating its superiority over 694 nm ruby and 1064 nm Nd:YAG lasers through direct comparative testing.

A 26-year-old woman developed extensive brownish hyperpigmentation following a ferric carboxymaltose infusion. She experienced immediate burning, swelling, and subsequent discoloration extending from her elbow to her wrist. Diagnosis of cutaneous siderosis was confirmed through clinical evaluation. Treatment involved four sessions of Q-switched Nd:YAG laser therapy at a 532 nm wavelength, based on the previous comparative findings. Treatment parameters ranged from 0.6 J/cm² to 1.0 J/cm² fluence with a 4 mm spot size. Clinical outcomes were assessed using a systematic approach with digital photographs and the Global Aesthetic Improvement Scale (GAIS). Gradual pigment reduction was observed throughout the treatment course, with complete resolution of hyperpigmentation achieved two years after the first treatment session.

Q-switched Nd:YAG laser therapy at 532 nm is an effective treatment for cutaneous siderosis resulting from iron infusion extravasation. This case demonstrates the potential for successful removal of iron-induced skin pigmentation with minimal adverse effects, offering a viable solution for patients experiencing this complication.

## Introduction

Iron, a vital component of hemoglobin and numerous enzymes, plays a crucial role in oxygen transport and cellular metabolism. Its deficiency can lead to a spectrum of clinical manifestations, ranging from fatigue and cognitive impairment to severe anemia. Women, particularly during periods of increased physiological demand such as pregnancy and the postpartum phase, are disproportionately affected by iron deficiency. When oral iron supplementation proves inadequate or unsuitable due to gastrointestinal intolerance or malabsorption, parenteral iron therapy is a suitable alternative intervention [1].

While effective in replenishing iron stores, parenteral iron administration is not without potential complications. Inadvertent extravasation of iron solutions, whether from intramuscular injections or intravenous infusions, can lead to the deposition of iron pigments in the surrounding tissues. This results in grayish-black to brown skin discoloration, which can be localized or widespread, as iron pigments can migrate from the site of injection and diffuse through the subcutaneous tissue, affecting adjacent skin areas [2]. These pigmentary changes can manifest days or weeks following the administration of iron and may persist for extended periods, and, in some cases, may become permanent. The resulting dyschromia can significantly impact a patient's quality of life, leading to emotional distress and social discomfort. Despite the potential for significant aesthetic and psychological distress, these complications are often underreported and insufficiently documented in the medical literature.

The management of iron-induced skin pigmentation presents a clinical challenge, primarily due to the lack of standardized treatment protocols and the limited understanding of the long-term behavior of deposited iron pigments. Given the paucity of evidence-based strategies for addressing this complication, this case report aims to contribute to the existing body of knowledge by presenting a successful treatment approach utilizing the 532 nm Q-switched Nd:YAG laser.

## Case presentation

Selection of laser parameters

Prior to developing our treatment protocol for extensive cutaneous siderosis, we conducted a comparative wavelength study to determine the optimal laser parameters. In this preliminary investigation, a 39-year-old patient with a Fitzpatrick III skin type presented with diffused pigmentary dermopathies, predominantly localized to the right leg (specifically on the medial aspect) and along the course of dilated veins, as a result of iron extravasation due to venous insufficiency. This condition, of recent onset (approximately six months), manifested as a brownish-ochre discoloration of the skin due to hemosiderin accumulation from hemoglobin catabolism, following micro extravasations of erythrocytes. To evaluate the efficacy of different laser wavelengths, three adjacent test areas were treated with distinct Q-switched laser wavelengths: 694 nm ruby laser, 532 nm Nd:YAG laser, and 1064 nm nanosecond Nd:YAG laser. For each wavelength, four test spots were created using increasing fluences (0.5 J/cm², 0.8 J/cm², 1 J/cm², and 1.2 J/cm²) to ensure comparable assessment conditions.

**Figure 1:**
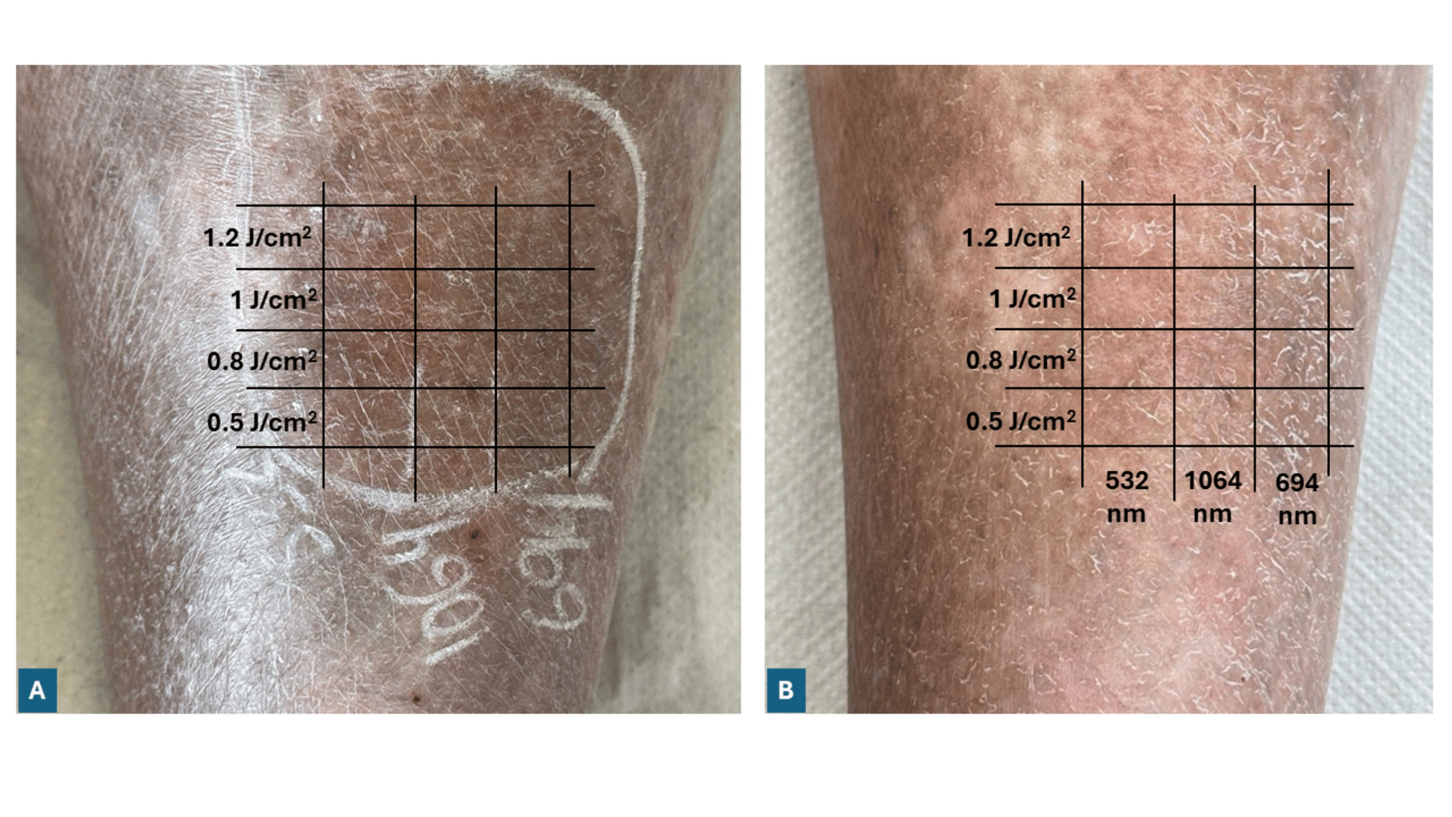
Test spots for comparative Q-switched laser treatment on the right leg of a 39-year-old woman with iron-induced hyperpigmentation A) Three Q-switched laser systems, a 532 nm Nd:YAG laser, a 1064 nm Nd:YAG laser, and a 694 nm Ruby laser were applied at increasing fluences (0.5 J/cm², 0.8 J/cm², 1 J/cm², and 1.2 J/cm²). B) One month after the test areas were treated, an improvement in hyperpigmentation was noticeable exclusively within the treatment spots corresponding to the 532 nm Nd:YAG laser applied at fluences of 1 J/cm² and 1.2 J/cm^2^. No significant changes were observed in the other test areas.

After a period of 35 days, a discernible improvement in hyperpigmentation was evident exclusively in the test area treated with the 532 nm nanosecond Nd:YAG laser (Figure 1). No significant improvement was observed in the areas treated with either the 694 nm ruby laser or the 1064 nm Nd:YAG laser, despite using similar energy parameters. This comparative assessment provided crucial evidence for wavelength-specific efficacy in iron pigmentation removal, definitively guiding our therapeutic approach toward selecting the 532 nm wavelength for subsequent clinical applications.

These findings directly informed our treatment strategy for the following complex case of extensive iatrogenic cutaneous siderosis.

Case study

A 26-year-old woman with a Fitzpatrick III skin type and a history of iron deficiency anemia presented with significant cutaneous siderosis following intravenous ferric carboxymaltose infusion (Ferinject 50 mg iron/injectable solution/infusion Vifor Pharma Italia s.r.l, dissolved in 250 mL 0.9% saline). Immediately following the infusion, the patient reported a burning sensation, localized swelling, and the rapid onset of brownish hyperpigmentation at the injection site. Within days, the swelling persisted, and the affected arm displayed a bruised appearance, functional movement limitations, and sensory deficits, accompanied by a fever of 39°C. Initial symptomatic treatment with mild corticosteroids, non-steroidal anti-inflammatory drugs (NSAIDs), and antipyretics resolved the acute symptoms, but left a persistent 20 cm x 40 cm area of brownish hyperpigmentation encircling the forearm from the elbow crease to the wrist.

The patient was subsequently referred for dermatological evaluation, where a diagnosis of iatrogenic cutaneous siderosis was confirmed. Based on the findings from our previous comparative test, which demonstrated superior efficacy of the 532 nm wavelength for iron pigmentation removal, we selected Q-switched Nd:YAG laser therapy (Discovery Pico Plus; Quanta System, Samarate, Varese, Italy) utilizing the 532 nm wavelength as the primary treatment modality.

Treatments were conducted with a fluence of 0.6 J/cm²-1.0 J/cm², a frequency of 1Hz, and a 4 mm spot size, without topical anesthesia. A total of four laser sessions were performed over 12 months, with the second session performed approximately one month after baseline, and subsequent sessions at approximately three-month intervals. Treatment progression was monitored through clinical observations and digital photographs, with the final follow-up assessment conducted two years after the initial treatment. The primary variables evaluated were the degree of hyperpigmentation reduction and the presence of adverse effects. The satisfaction rate was evaluated by the physician and the subject based on the Global Aesthetic Improvement Scale (GAIS) assessment on a 5-point scale from “worse” to “very much improved”. The improvement scale with reference to the baseline condition was assessed independently by both subject and physician at every follow-up visit.

**Figure 2:**
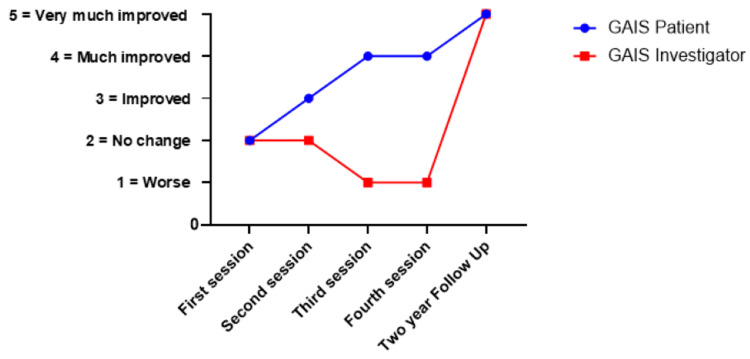
GAIS assessment by physician and patient over time GAIS: Global Aesthetic Improvement Scale

**Figure 3:**
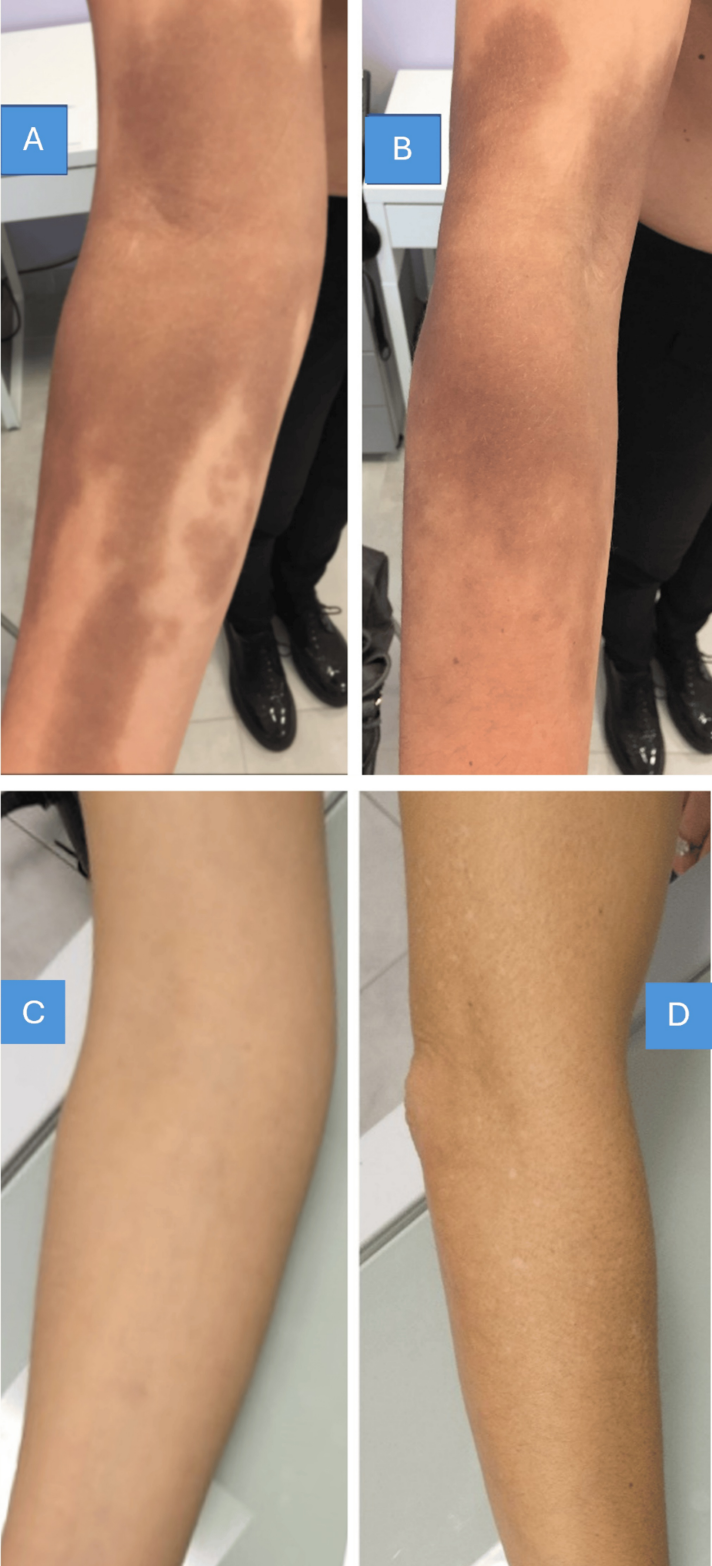
Clinical presentation of cutaneous siderosis before and after treatment with a Q-switched 532 nm Nd:YAG laser A. Initial clinical presentation, anterior side of the limb. B. Initial clinical presentation, lateral side of the limb. C. Clinical response 12 months after the completion of laser treatment, anterior side. D. Clinical response 12 months after the completion of laser treatment, lateral side.

**Figure 4:**
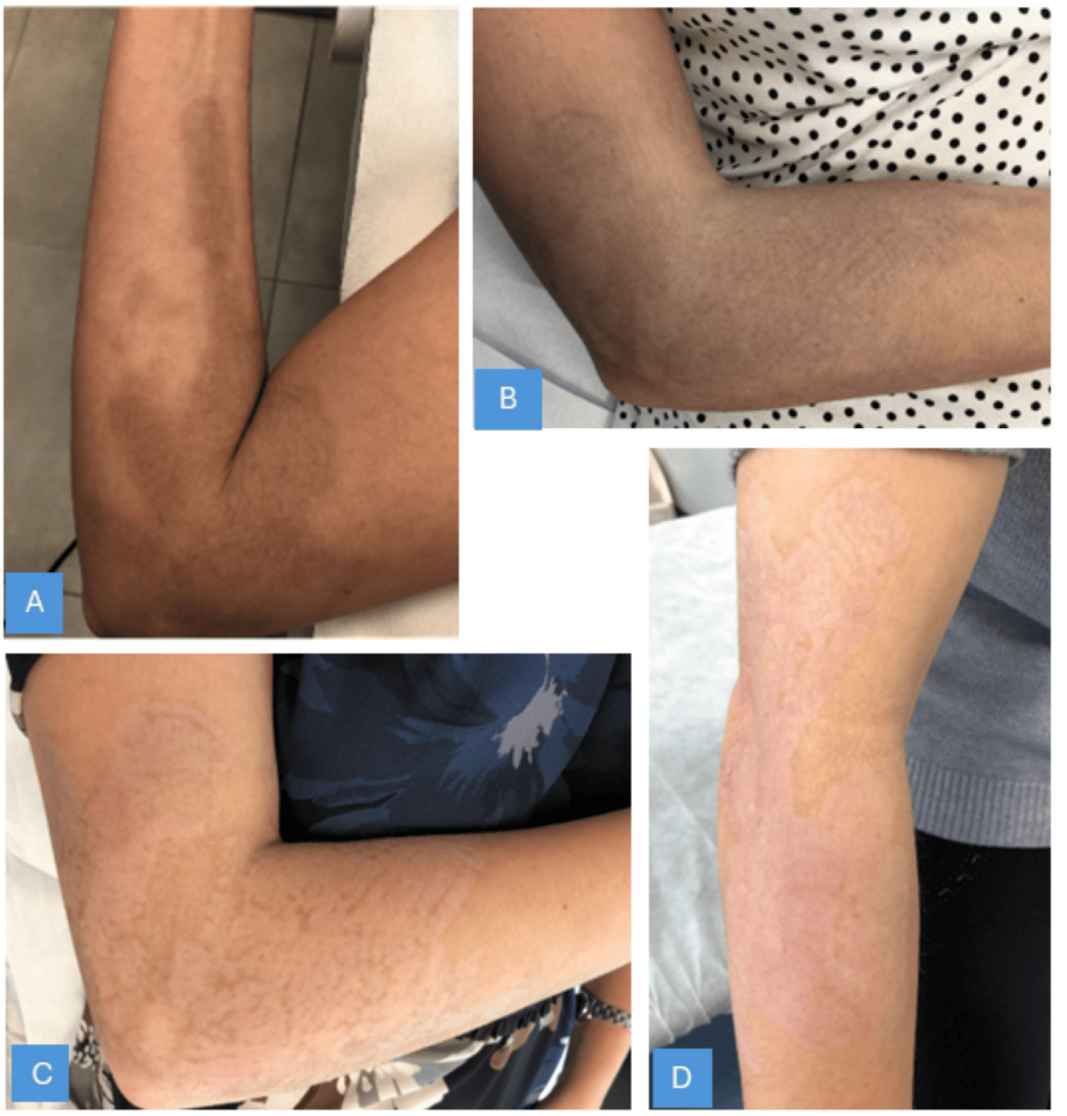
Clinical progression during Q-switched 532 nm laser treatment of cutaneous siderosis Clinical evolution after the first (A), second (B), third (C), and final session (D). Note the development of transient hypopigmentation in images C and D, a temporary adverse event typical of laser treatment for pigmentary alterations.

Analysis of GAIS scores demonstrated a notable divergence between patient and investigator assessments during the treatment course. Patients consistently reported progressive improvement from the first session onwards; in contrast, the investigator's evaluation exhibited a characteristic biphasic pattern: an initial decline to "Worse" after the third and fourth sessions, followed by a dramatic improvement to "Very much improved" at the final follow-up (Figure 2). This temporary deterioration in the investigator's assessment reflected the development of post-inflammatory hypopigmentation, a recognized sequela of the treatment process that temporarily replaced the original cutaneous siderosis with a different aesthetic concern. Importantly, this hypopigmentation represents an expected treatment response indicating active therapeutic engagement rather than treatment failure. By the final assessment, both patient and investigator evaluations converged at "Very much improved," confirming complete resolution of the cutaneous siderosis, defined as no clinically visible traces of the original pigmentation, and restoration of normal skin appearance (Figure 3 and Figure 4).

The treatment was well-tolerated, with mild post-treatment crusting and purpura resolving within two weeks. No other persistent side effects such as scarring or burns occurred.

The patient reported significant improvement in her quality of life and satisfaction with the treatment outcome. The successful management of this challenging case of extensive cutaneous siderosis corroborated the initial wavelength selection findings and established a promising treatment protocol for similar cases of iron-induced pigmentation.

## Discussion

Extravasation, the inadvertent leakage of intravenous solutions into surrounding tissues, is a significant clinical concern. While particularly dreaded in the context of anti-cancer chemotherapy due to the risk of severe skin necrosis, extravasation of iron solutions is often considered less critical, primarily resulting in skin pigmentation from iron deposition within the subcutaneous tissue. However, despite this perception, the long-term persistence of such pigmentation remains poorly understood, and standardized management protocols are lacking.

The challenge of treating persistent pigmentation caused by metallic salt deposition in the cutaneous connective tissue is well-recognized. Conditions such as exogenous ochronosis, chrysiasis, argyrosis, and siderosis, including accidental tattoos, illustrate the difficulties in addressing exogenous dyschromia [3]. Although localized hyperpigmentation at the injection site is well-documented following iron infusions, our case and others reveal the potential for more widespread pigmentation, highlighting the need for careful administration and monitoring.

Histologically, cutaneous siderosis is characterized by iron deposits within macrophages, appearing as brown granules that stain blue with Prussian blue. Notably, these iron-laden macrophages are distributed throughout the dermal layers, with a marked concentration around vascular structures (perivascular) and skin appendages (periadnexal), particularly eccrine sweat glands. Furthermore, the deposits extend into the subcutaneous tissue, reaching depths of 5 mm to 7 mm [4-6]. This contrasts with decorative tattoos, where pigment granules are primarily located in the upper dermis [7].

The aesthetic burden of iron-induced pigmentation necessitates effective treatment strategies. Q-switched laser systems, including ruby, Nd:YAG, and alexandrite lasers, have demonstrated efficacy in removing various pigment deposits, including tattoos [8].

This efficacy is rooted in the principle of selective photothermolysis, pioneered by Anderson and Parrish [9], which forms the fundamental basis for laser-based pigment removal. QS lasers, delivering nanosecond pulses, effectively fragment pigment particles through photothermal and photoacoustic effects, facilitating macrophage phagocytosis and lymphatic clearance. Picosecond (PS) lasers, with shorter pulse durations, offer a potentially more efficient approach through a stronger photoacoustic effect, although their superiority over QS lasers in tattoo removal remains debated [10-12]. Both QS and PS lasers have been employed to treat iron-induced pigmentation with variable success, and a definitive treatment protocol remains to be established, given that current research is primarily comprised of case reports and case series.

Early evidence regarding the efficacy of Q-switched lasers in treating cutaneous iron deposits dates back to 2001, when Raulin et al. retrospectively analyzed data from five female patients treated with QS ruby (694 nm) and/or Nd:YAG (1064 nm) lasers. The number of laser treatments administered ranged from three to 16 per patient, with intervals of four to eight weeks. Energy densities employed were between 6 J/cm^2^ and 40 J/cm^2^ for the ruby laser and between 3.5 J/cm^2^ and 6.1 J/cm^2^ for the Nd:YAG laser. A visible lightening of the brownish-gray dyschromia was achieved in all cases, but none of the patients had complete resolution of the pigmentation [5]. It is interesting to note that, while Raulin's study demonstrated partial efficacy with both 694 nm and 1064 nm wavelengths, our comparative assessment revealed clinical improvement exclusively with the 532 nm Nd:YAG laser. This specificity suggests that a 532 nm wavelength may provide optimal absorption characteristics for hemosiderin deposits in cutaneous siderosis, potentially offering superior targeting compared to the 694 nm and 1064 nm options.

Fourteen years later, two case studies highlighted the potential of 755 nm alexandrite Q-switched lasers as an effective treatment modality for cutaneous siderosis. The first case involved a 68-year-old female patient with a yellow-brown patch on the left antecubital fossa resulting from an iron infusion extravasation six months prior. After two treatment sessions with a 755 nm laser (initially 10 J/cm² and subsequently 13 J/cm²), the patient showed a dramatic decrease in pigmentation within two months. This outcome was attributed to the effective energy absorption by hemosiderin granules, which exhibit absorption peaks at 470 nm-480 nm and 660 nm-680 nm, allowing the 755 nm laser to mechanically destroy the iron deposits in the dermis [13]. Similarly, Lloyd et al. documented the successful treatment of hyperpigmentation that developed in a 50-year-old patient after intramuscular iron dextran injections with four sessions of 755 nm Q-switched laser. The authors noted the laser's capacity for deep dermal-subcutaneous penetration, which allowed for precise targeting of iron deposits while minimizing the potential for epidermal injury [14].

The use of multiple laser wavelengths demonstrated enhanced efficacy in the treatment of accidental hyperpigmentation resulting from iron injections. In a cohort of 13 patients treated with QS ruby, QS Nd:YAG, and PS Nd:YAG lasers, complete clearance was observed in eight individuals after an average of 5.6 treatment sessions. The authors emphasized the influence of Fitzpatrick skin phototype on wavelength selection and recommended a conservative fluence approach with the QS ruby laser in patients with higher phototypes to minimize adverse effects [15]. 

A 2020 study by Heidemeyer et al. confirmed effectiveness ofQ-switched Nd:YAG (1064nm, 532nm) and ruby (694nm) lasers for cutaneous siderosis in a population of 15 female patients. Most patients achieved significant pigment clearance, evaluated using a 5-point Physician Global Assessment (PSA) scale, with an average of 4.5 sessions (range two to nine). Side effects were limited to a mild burning sensation and mild transient purpura. Different wavelengths of Q-switched lasers appeared beneficial, as both the depth of penetration (greater with longer wavelengths) and the iron absorption peak (415 nm) are important [16].

Although existing literature suggests superior efficacy for PS lasers over QS lasers in tattoo removal, and given the histological similarities between cutaneous siderosis and decorative tattoos, this superiority has not been consistently observed in siderosis treatment [17]. A split-lesion trial directly comparing the efficacy of PS and QS 1064nm lasers on a single patient reported marginally greater clearance with QS 1064nm laser, while Lee et al. documented the need for ten PS 1064nm laser sessions to achieve significant improvement [6,16].

This case report describes the effective treatment of iatrogenic cutaneous siderosis post-intravenous ferric carboxymaltose infusion with a 532 nm Q-switched Nd:YAG laser. This case, involving significant hyperpigmentation in a 26-year-old woman, adds to the growing literature on the use of lasers in treating this condition.

Similar to the case presented by Hammami Ghorbel [18], our study demonstrated the efficacy of the 532 nm Nd:YAG laser in reducing iron-induced hyperpigmentation. However, there are some notable differences. The Hammami Ghorbel case involved an older patient (65 years old) with chronic anemia treated with iron sucrose, and the hyperpigmentation resulted from paravenous extravasation. Our case, on the other hand, involved a younger patient (26 years old) with iron deficiency anemia, treated with ferric carboxymaltose, and the hyperpigmentation developed immediately after the infusion. Furthermore, Hammami Ghorbel and colleagues used a 532 nm Nd:YAG laser with higher fluences (4.4 J/cm²) and smaller spot sizes (2 mm), requiring topical anesthesia. Our study used lower fluences (0.5-1.0 J/cm²) and larger spot sizes (4 mm-5 mm), without topical anesthesia, still achieving an effective result with the same four-session treatment protocol.

The study by Sharma et al. [19], which describes a 41-year-old patient with Crohn's disease treated with ferric carboxymaltose, shares similarities with our case regarding the type of iron administered. However, the authors used a slightly different treatment protocol, with fluences of 1 J/cm²-3 J/cm² and spot sizes of 3.4 mm-4.3 mm, achieving a positive result after three sessions.

A notable difference between the cases lies in the extent of the hyperpigmented area. Our case presented with a particularly extensive lesion, measuring 20 cm x 40 cm and encircling the entire forearm. In the Hammami Ghorbel case, the hyperpigmented area was smaller, measuring 10 cm x 15 cm and localized to the forearm. In Sharma’s case, the lesion size was not explicitly quantified.

Furthermore, a significant difference between our case report and the other two studies lies in the documentation of adverse events and the approach to evaluating results. In our case and in the Hammami Ghorbel study, transient side effects, such as crusting and purpura, which resolved spontaneously within two weeks, were reported. In the case report by Sharma et al., instead, adverse events are not mentioned.

Moreover, our study employed the GAIS, an objective scale, to quantify aesthetic improvement and patient satisfaction. This scale was not used in the other two studies, which relied mainly on clinical observations and photographs.

The observed discrepancy between patient and physician GAIS assessments in this study highlights a key aspect of treatment perception. The physician “worse” score after the third and fourth sessions reflected the development of post-inflammatory hypopigmentation, an expected, temporary side effect of the treatment process. From the physician's perspective, these hypopigmented patches created a different type of dyschromia, which was perceived as a deterioration from the baseline condition.

Conversely, the patient consistently reported progressive improvement from the first session onward. The patient's focus was on the reduction of the highly visible hyperpigmentation, which was the primary source of her distress. The lightening of the original dark pigmentation was therefore perceived as a significant improvement in the overall condition, regardless of the temporary hypopigmentation.

The eventual convergence of both assessments at "Very much improved" at final follow-up validates the treatment efficacy and demonstrates that the intermediate hypopigmentation was indeed a transient phenomenon. This pattern underscores the importance of comprehensive patient counselling regarding expected treatment phases, including the possibility of temporary aesthetic worsening before ultimate improvement. Furthermore, it highlights the value of utilizing both patient-reported and physician-assessed outcomes to fully capture the success of aesthetic treatments.

## Conclusions

This case report demonstrates that Q-switched Nd:YAG laser therapy at 532 nm represents an effective and well-tolerated treatment option for cutaneous siderosis. Our comparative wavelength evaluation definitively established the superior efficacy of 532 nm over both 694 nm ruby and 1064 nm Nd:YAG wavelengths for iron pigmentation removal, providing valuable guidance for clinical decision-making. The successful treatment of extensive hyperpigmentation with complete resolution achieved after four sessions and maintained at two-year follow-up underscores the therapeutic potential of this approach. Notably, the development of transient hypopigmentation observed during treatment represents an expected therapeutic response rather than treatment failure, given its temporary nature and subsequent complete resolution to normal skin appearance. These findings contribute to the limited but growing evidence base for laser management of iron-induced skin pigmentation and support the establishment of 532 nm Q-switched Nd:YAG laser as a first-line treatment option. Further prospective studies with larger patient cohorts are warranted to validate these findings and establish standardized treatment protocols for this challenging aesthetic complication.
